# The immunological characteristics of TSPAN1 expressing B cells in autoimmune hepatitis

**DOI:** 10.3389/fimmu.2022.1076594

**Published:** 2022-12-15

**Authors:** Yiyan Ou, Ruiling Chen, Qiwei Qian, Nana Cui, Qi Miao, Ruqi Tang, Zhengrui You, Xiong Ma, Qixia Wang

**Affiliations:** ^1^ Division of Gastroenterology and Hepatology, Key Laboratory of Gastroenterology and Hepatology, Ministry of Health, State Key Laboratory for Oncogenes and Related Genes, Renji Hospital, School of Medicine, Shanghai Institute of Digestive Disease, Shanghai Jiao Tong University, Shanghai, China; ^2^ Division of Infectious Diseases, Renji Hospital, School of Medicine, Shanghai Jiao Tong University, Shanghai, China

**Keywords:** autoimmune hepatitis, tetraspanin 1, B cells, cytokines, antigen presentation

## Abstract

**Background and aims:**

Tetraspanin proteins are closely related to the functional changes of B cells, including antigen presentation, production of cytokines, and transduction. We aim to explore the potential role of Tetraspanin 1 (TSPAN1) in the biological activities of B cells in AIH.

**Methods and results:**

Herein, this study found that numbers of cells expressing TSPAN1 were significantly increased in AIH patients compared to PBC, chronic hepatitis B, and healthy control (P < 0.0001). Moreover, there was a positive correlation between numbers of TSPAN1+ cells and AIH disease severity (P < 0.0001). Immunofluorescence staining further confirmed that TSPAN1 was primarily expressed on CD19+ B cells. Flow-cytometric analysis showed that TSPAN1+ B cells secreted more inflammatory cytokines and expressed higher level of CD86 than TSPAN1- B cells. Furthermore, compared with TSAPN1- cells, the expression of CXCR3 on TSPAN1+ cells was also higher. Meanwhile, CXCL10, the ligand of CXCR3, was significantly elevated in the liver of AIH (P < 0.01) and had positive correlation with the quantities of TSPAN1 (P < 0.05). Interestingly, the numbers of TSPAN1+ B cells were decreased in AIH patients after immunosuppressive therapy.

**Conclusions:**

TSPAN1^+^ B cells in the liver may promote the progression of AIH *via* secreting cytokines and presenting antigens. The chemotactic movement of TSPAN1^+^ B cells toward the liver of AIH was possibly due to CXCR3 - CXCL10 interaction.

## 1 Introduction

Autoimmune hepatitis (AIH) is recognized as an immune mediated disease, characterized by hypergammaglobulinemia, female preponderance and liver-infiltrating immune cells in portal area (1, 2). The mechanisms underlying the breakdown of self-tolerance have not been fully elucidated, though there is mounting evidence that autoreactive T cell and dysfunction of regulatory T cells play a key role (3). However, anti-CD20 may be a useful treatment for patients with a poor response to conventional therapy, indicating a vital role of B cells in AIH (4, 5).

Previous studies have shown that B lymphocytes can be involved in the development of AIH. Autoreactive B cells are typically considered as the sources of autoantibodies. Seropositivity to specific autoantigens is used for distinguishing two types of AIH. Additionally, hypergammaglobulinemia with specific immunoglobulin G (IgG) is another characteristic diagnostic hallmark of AIH, which has been demonstrated to correlate with disease activity (6, 7). Moreover, B lymphocytes can drive autoimmunity as antigen presenting cells (APCs). In the absence of other APCs, activation of CD4^+^ T cells and T follicular helper (Tfh) can be initiated by B cells. It has been reported that B cell-derived cytokines have double-edged effects on AIH (8). On one hand, pro-inflammatory cytokines like type I interferons, tumor necrosis factor-alpha (TNF-α), and interleukin (IL) 6 contribute directly or indirectly to disease progression. On the other hand, B cells can also secret IL-10 and transforming growth factor-beta (TGF-β) to induce Tfh and IL-10 producing T cells (9).

Tetraspanins are highly conserved proteins with four transmembrane domains, and known to play an important role in B and T lymphocytes (10), including cell proliferation, motility, and adhesion. For example, CD9, CD53, CD81, and CD82 all belong to the tetraspanins family, which can bind to CD19, CD21, and HLA-DR expressed on mature B cells, and are tightly associated with the functional changes of cells (11, 12). Specially, CD9, CD151, and CD81 also contribute to antigen presentation, production of cytokines, and signal transduction (13). Tetraspanin 1 (TSPAN1) as a member of tetraspanin superfamily, is mainly expressed on plasma membrane and intracellular vesicles (14). TSPAN1 has been reported to involve in a variety of biological functions, including cell proliferation, adhesion, and migration (15). It has been found that TSPAN1 might be related to poor prognosis of pancreatic cancer and ovarian cancer (16, 17). In addition, TSPAN1 silence also has effects on reducing the ratio of CD4^+^ T cells polarizing to Th17 cells (18). However, the function of TSPAN1 on B cells remains unclear.

Herein, we reported that TSPAN1 expression was significantly elevated in the liver of AIH patients, and positively correlated with disease severity. Confocal staining further confirmed that the majority of TSPAN1^+^ cells were CD19 positive in AIH. Interestingly, TSPAN1^+^ B cells had stronger ability to present antigens and secret cytokines compared with TSPAN1^-^ cells. Furthermore, CXCR3-CXCL10 interaction may be involved in the chemotaxis of TSPAN1^+^ B cells to the liver.

## 2 Materials and methods

### 2.1 Liver samples

Liver samples were obtained from 66 patients diagnosed as AIH, 24 patients with primary biliary cirrhosis (PBC), 21 chronic hepatitis B (CHB), and 7 healthy controls (HC). All patients corresponded to diagnosis standards of AIH (19), PBC (20), and CHB (21). Additionally, 27 of the patients with AIH had a follow-up biopsy to explore whether they had histological remission after three years of standard immunosuppressive treatment. Another 28 AIH patients with secondary liver biopsy were enrolled to further investigate the relationship between histologic remission and the frequency of TSPAN1. Immunohistochemical liver tissues from patients with AIH, PBC, and CHB were derived from ultrasound-guided needle liver biopsies. Besides, the liver tissue activity was assessed by the Scheuer scoring system for inflammation and fibrosis stages (22). AIH patients were divided into histologic remission and non-remission according to the Histological Activity Index (HAI) scoring system (23). Liver samples of HC were collected from explant donors before transplantation. Peripheral blood mononuclear cells (PBMCs), from patients who met AIH diagnostic criteria, were separated and frozen in fetal bovine serum (FBS) with 10% dimethyl sulphoxide. PBMCs were used to investigate the phenotypes and functions of TSPAN1^+^ B cells. The demographic and clinical features of these subjects were listed in **Table 1** and **Table 2**.

**Table 1:** Clinical Features of Patients with AIH, PBC, CHB, and HC.

	AIH(diagnostic biopsy) (n = 66)	PBC(n = 24)	CHB(n = 21)	HC(n = 7)	AIH(follow-up biopsy)(n = 55)
**Age (years)**	46.3 ± 12.3	44.2 ± 9.3	39.6 ± 14	36 ± 11.1	48.3 ± 10.9
**Gender (F/M)**	57/9	19/5	9/12	3/4	49/6
**ALT(U/L)**	154.6 ± 136.3	50.9±32.9	69.6 ± 118.9	33.7 ± 26.3	15.1 ± 9.4
**AST(U/L)**	144.3 ± 160.3	40.3±20.7	42.3± 52.6	26.1 ± 6	17.7 ± 5.2
**ALP(U/L)**	111.8 ± 72	166.8±103.5	81 ± 21.4	85.1 ± 25.6	58.7 ± 16.6
**GGT(U/L)**	117.1 ± 127.9	113.8±112.7	32.4 ± 19.1	21.3± 12.6	18.7 ± 14.6
**TBIL(μmol/L)**	47 ± 107.4	11.7±4.2	17.7 ± 23.1	11.9 ± 3.8	10.4 ± 5.8
**IgG(g/L)**	17.4 ± 5.8	13.8±4	12.3 ± 3.9	\	11.9 ± 2.3

**Table 2:** Clinical Features of Patients with AIH and HC.

	AIH (n = 14)	HC c(n = 10)
**Age (years)**	50.4 ± 10.8	50 ± 13.3
**Gender (F/M)**	9/5	8/2
**ALT(U/L)**	69.1 ± 45.3	20.2 ± 9.1
**AST(U/L)**	60.9± 54.3	20.7 ± 3.8
**ALP(U/L)**	110.5 ± 23	63.8 ± 9.7
**GGT(U/L)**	72.6 ± 85.6	15.3 ± 7.1
**TBIL(μmol/L)**	15.3 ± 9.9	10.3 ± 3.8
**IgG(g/L)**	17.5 ± 7.1	\

### 2.2 Immunohistochemistry

Formalin-fixed, paraffin-embedded liver tissues were prepared for immunohistochemistry. The procedures for immunohistochemistry had been described in the previous study (24). Concisely, liver sections were blocked with goat serum for 30 minutes and then incubated with primary antibody TSPAN1 (GTX108675, GeneTex) or CXCL10 (AF-266-NA, R&D systems) overnight at 4°C. After washing in phosphate buffered saline, the incubation with a horseradish peroxidase-conjugated secondary antibody was performed at room temperature for 30 minutes. Lastly, the nuclei were stained by 3,3′-diaminobenzidine and hematoxylin. The numbers of positive sections were calculated blindly by two independent observers. Five fields of portal areas were selected stochastically from each liver section. Numbers of infiltrative TSPAN1^+^ cells were acquired per high-power field. Score CXCL10 expression from zero to four points per high power field. Cases were scored at one if the expression area < 25% and two if ≥ 25% to <50%, three if ≥ 50% to < 75%, four if ≥ 75%.

### 2.3 Confocal staining

Procedures for confocal laser scanning microscopy had been described before (25). After formalin-fixation, paraffin embedding, the liver samples were incubated in 3% H_2_O_2_ for 10 minutes and goat serum for 30 minutes successively at room temperature, then washed in phosphate buffer saline. Next, the sections were incubated with two primary antibodies overnight at 4°C after undergoing antigen retrieval. The samples were incubated with AF488 and AF555 fluorochrome-conjugated secondary antibodies (1:500; Invitrogen, Carlsbad, CA) for 30 minutes. The nuclei were stained by DAPI (Southern Biotech, Birmingham, AL). The confocal scanning was performed by LSM-710 laser-scanning confocal microscope (Carl Zeiss, Jena, Germany).

### 2.4 Multiplex immunofluorescence staining

Multiplex immunofluorescence staining was performed by the Opal 4-Color IHC kit (Abs50028-20T, Absin). The liver sections were processed by antigen retrieval. The primary antibody of TSPAN1 was incubated at 37°C for 1 hour. Then, the sections were washed and incubated with HRP-conjugated secondary antibody for 20 minutes at room temperature, what followed was adding TSA dye 520. The second antibody CD19 (ab134114, Abcam) was incubated overnight at 4°C, what followed was adding TSA dye 650. The last antibody CXCR3 (ab288437, Abcam) and TSA dye 570 were added sequentially. The nuclei were stained with DAPI.

### 2.5 *In vitro* culture

PBMCs were isolated from fresh blood through gradient centrifugation using Ficoll Hypaque Plus (GE Healthcare). CD19^+^ B cells were further separated from PBMCs by using CD19 Microbeads (130-097-055 Miltenyi Biotec). CD19^+^ B cells at 1 × 10^5^ cells/mL were cultured in 96-well culture plates, where the complete medium consists of the Roswell Park Memorial Institute 1640 (RPMI-1640), 10% heat-inactivated fetal bovine serum, 100 U/mL penicillin, 100 ug/mL streptomycin, and 50 mM 2-mercaptoethanol. To stimulate B cells, 2 μg/mL Cytidine-phosphate-guanosine (CPG) (Invitrogen, Carlsbad, CA), 1 μg/mL CD40 ligand (CD40L) (PeproTech, Cranbury, NJ), 50ng/mL rhIL-4 (PeproTech, Cranbury, NJ) were added in complete medium. Cells were collected after 48 hours.

### 2.6 Transwell assay

CD19^+^ B cells were isolated from PBMCs of AIH patients and cultured in 96-well culture plates with CPG, CD40L, and IL-4 for 24 hours. Then, cells were harvested and placed on the upper chamber of 24-well transwell plate with 5 μm pores (Coring, Kennebunk, MA, 3421), where consist of the complete medium for 200 μL. In the lower chamber, rhCXCL10 was added with 3 μg/mL in the complete medium, while the complete medium was as the control group. Cells in the lower chamber were harvested and analyzed after 6 hours.

### 2.7 Flow-cytometric analysis

The frozen PBMCs were resuscitated, washed and labeled with fluorochrome-conjugated antibodies, including anti-mouse TSPAN1, CD19, CD11c, CD38, CD80, CD86, HLADR, CXCR3, CXCR4 (BD Bioscience, San Diego, CA, USA). In intracellular cytokine staining, the cells were stimulated with lipopolysaccharide for 1 hour, and then Leukocyte Activation Cocktail (BD Biosciences, San Diego, CA, USA) for 4 hours at 37°C. Specific fluorochrome-conjugated antibodies were first added to stain surface markers at 4°C for 30 minutes. Subsequently, cells were fixed and permeabilized with Cytofix/Cytoperm solution (BD Biosciences, San Diego, CA, USA) at 4°C for 20 minutes. Ultimately, intracellular markers including granzyme B, interferon-γ (IFN-γ), TNF-α, and TGF-β (BD Biosciences, San Diego, CA, USA) were stained at 4°C for 1 hour. All cells were detected by LSR Fortessa X-20 analyzers (BD Biosciences). Statistics and *t*-SNE (*t*-distributed stochastic neighbor embedding) analysis were performed by FlowJo.

### 2.8 Statistical analysis

All statistical analysis were performed by GraphPad Prism 8.0 software. For normally distributed data, paired or unpaired Student *t*-test were used for comparisons between two groups. Statistical difference for abnormally distributed data were analyzed by Mann-Whitney U test. Wilcoxon matched-pairs test was used to analyze two paired groups. Correlation analyses were performed by Pearson’s correlation test. All analyses were two-tailed, and *P* < 0.05 was considered significant.

## 3 Results

### 3.1 TSPAN1 expression was increased in AIH and correlated with disease activity

**Figure 1:**
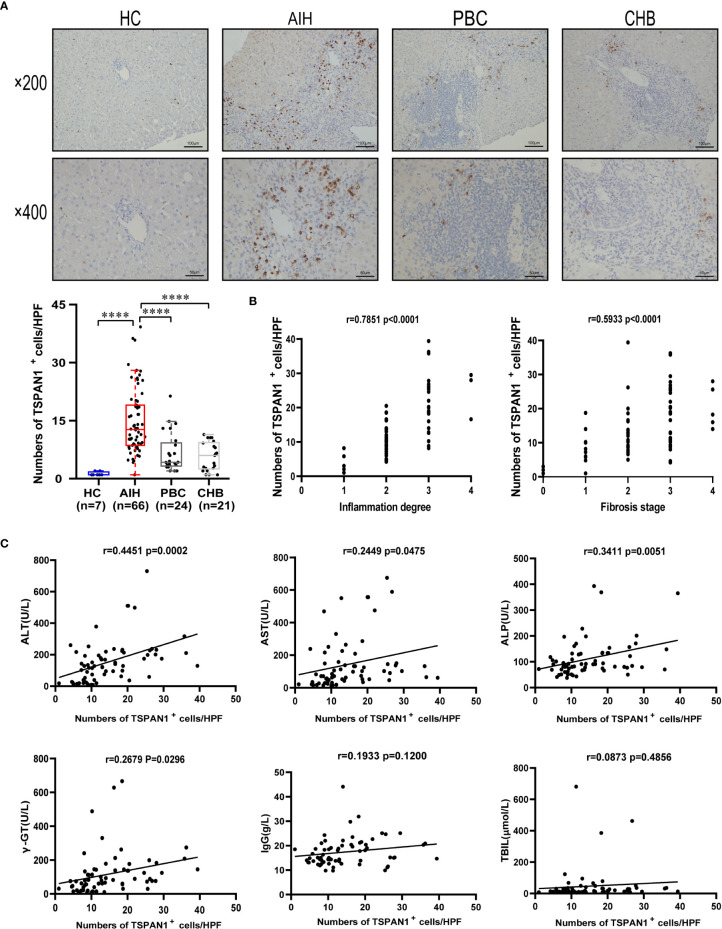
TSPAN1 expression was increased in AIH. **(A)** Representative immunohistochemical staining of TSPAN1 in AIH, PBC, CHB, and healthy controls (HCs). **(B)** The correlation analysis of the numbers of TSPAN1^+^ cells in liver tissues of AIH with hepatic inflammation degree and fibrosis stage (×400). **(C)** The correlation analysis of the numbers of TSPAN1^+^ cells in liver tissues of AIH with ALT, AST, ALP, γGT, IgG, and TBIL levels. ****P < 0.0001.

Immunohistochemical staining was performed in liver tissues of AIH (n = 66), PBC (n = 24), CHB (n = 21), and HC (n = 7), the numbers of TSPAN1^+^ cells were significantly increased in AIH compared to PBC (*P* < 0.0001), CHB (*P* < 0.0001), and HC (*P* < 0.0001) (**Figure 1A**). TSPAN1^+^ cells mainly accumulated in the portal tracts and few scatted in inter-lobular areas. To further explore the clinical significance of TSPAN1^+^ cells in AIH, the correlation between the numbers of portal TSPAN1^+^ cells with clinical and histological indices was analyzed. Interestingly, the frequency of TSPAN1^+^ cells was positively correlated with hepatic inflammation degree (r = 0.7851, *P* < 0.0001) and fibrosis stage (r = 0.5933, *P* < 0.0001) (**Figure 1B**). Moreover, there was a positive correlation of TSPAN1^+^ cells with serum alanine transaminase (ALT) levels (r = 0.4451, *P* = 0.0002), aspartate aminotransferase (AST) levels (r = 0.2449, *P* = 0.0475), alkaline phosphatase (ALP) levels (r = 0.3411, *P* = 0.0051) and gamma-glutamyltransferase (γ-GT) (r = 0.2679, *P* = 0.0296). However, no statistical correlation was observed between TSPAN1^+^ cells with serum IgG and total bilirubin (TBIL) levels (**Figure 1C**). These results indicated that TSPAN1^+^ cells may play an important role in the development of AIH.

### 3.2 TSPAN1 was mainly expressed on hepatic B cells in AIH

**Figure 2:**
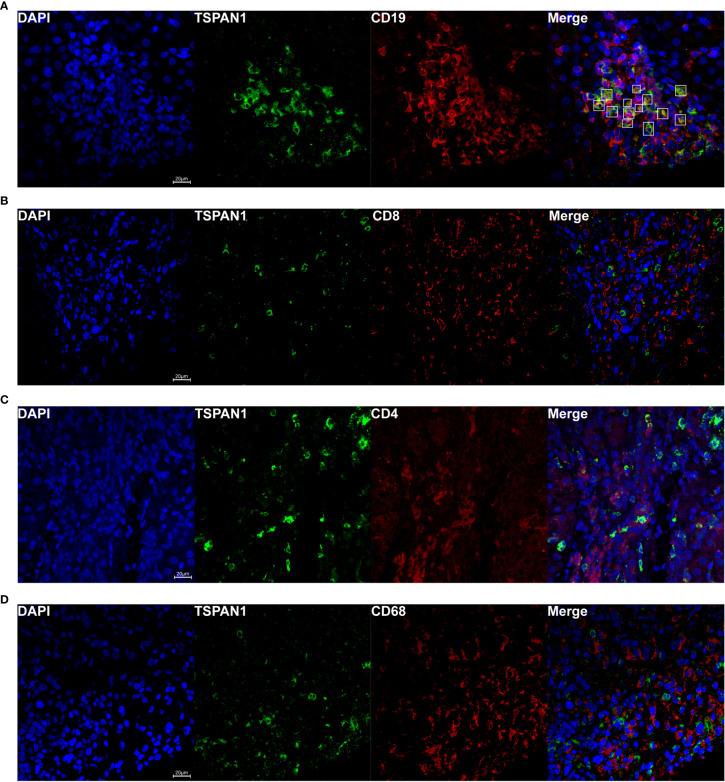
TSPAN1 was mainly expressed on hepatic B cells in AIH. Representative confocal staining of TSPAN1 with CD19 **(A)**, CD8 **(B)**, CD4 **(C)**, and CD68 **(D)** (×400) in liver tissues of AIH patients.

**Figure 3:**
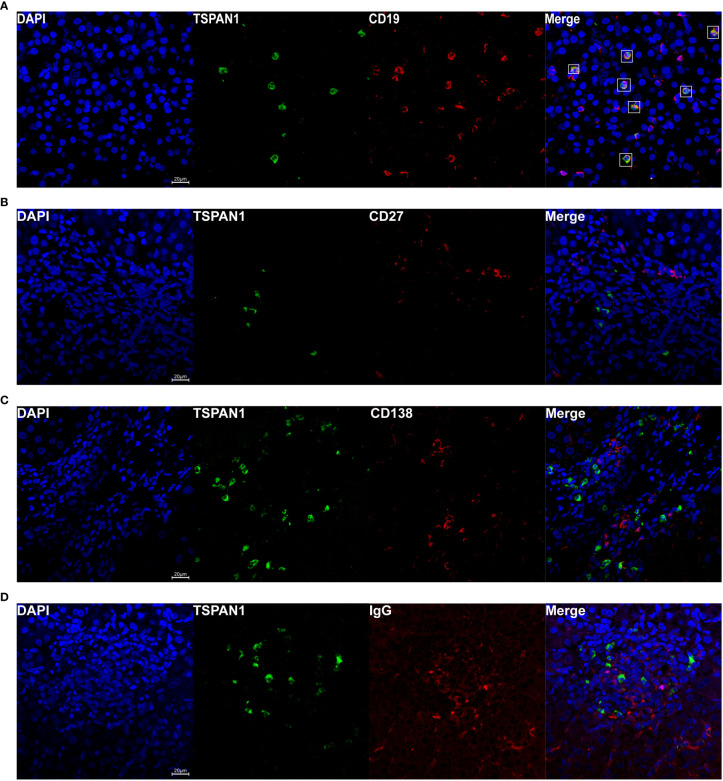
Immunofluorescence double-staining of CD19, CD27, CD138, and IgG with TSPAN1. Representative confocal staining of TSPAN1 with CD19 **(A)**, CD27 **(B)**, CD138 **(C)**, and IgG **(D)** (×400) in liver tissues of AIH patients.

To investigate which category of the cells expressed TSPAN1 in AIH, we co-stained TSPAN1 with CD19, CD8, CD4 or CD68 by immunofluorescence double-staining. As shown in **Figure 2A**, most of TSPAN1 positive cells were colocalized with CD19. However, TSPAN1 was less expressed on CD8, CD4, and CD68 cells (**Figure 2**). The results indicated that infiltrating TSPAN1^+^ cells in the liver of AIH were mainly B cells, rather than T cells or macrophagocytes. To further analyze the subpopulation of TSPAN1^+^CD19^+^ B cells in AIH, we co-stained TSPAN1 with CD27, CD138, IgG. Surprisingly, there was no co-expression between TSPAN1 and these plasma cell markers (**Figure 3**).

### 3.3 The immunological features of TSPAN1 expressing B cells in AIH

**Figure 4:**
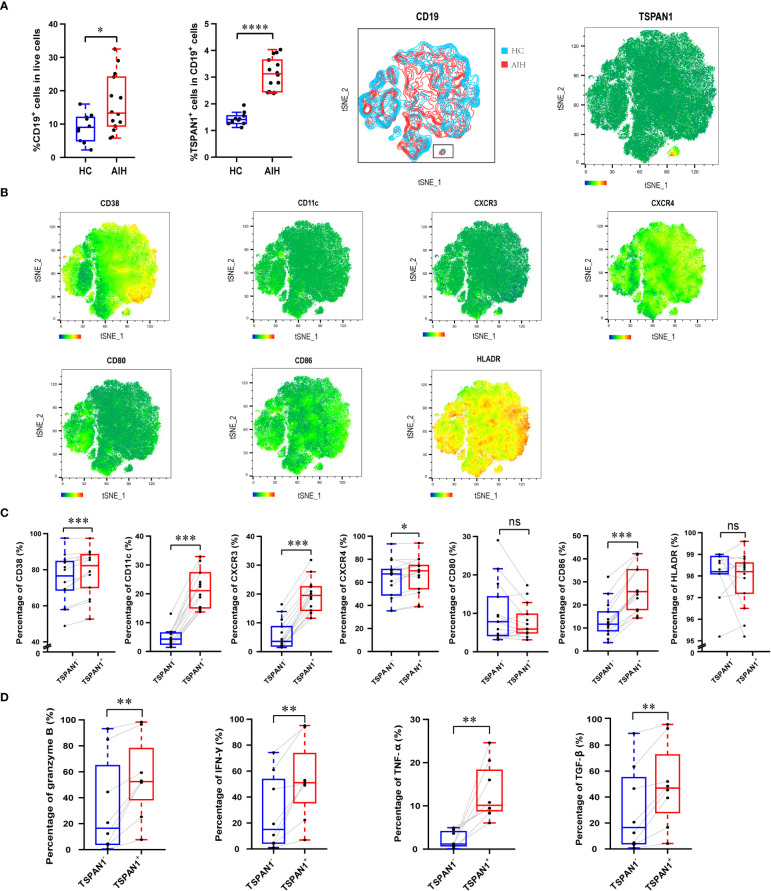
The immunological characteristics of TSPAN1^+^ cells in AIH. **(A)** The percentage of CD19^+^ B cells and TSPAN1^+^ cells in peripheral blood mononuclear cells were elevated in AIH patients (n = 14) compared with HCs (n = 10). By *t*-SNE analysis of CD19^+^ B cells, the TSPAN1^+^ cell subset was compared between HC and AIH indicated by the black rectangle (left), and TSPAN1 expression in CD19^+^ B cells was shown in AIH (right). **(B)** Expression of surface markers was shown by *t*-SNE analysis of CD19^+^ B cells from AIH patients. Color is based on the expression of certain markers. **(C)** Analysis of surface markers in paired TSPAN1^-^ cells and TSPAN1^+^ CD19^+^ cells from patients with AIH. Wilcoxon matched-pairs test was used. **(D)** Expression of cytokines in paired TSPAN1^-^ cells and TSPAN1^+^ CD19^+^ cells from patients. *P < 0.05, **P < 0.01, ***P < 0.001, ****P < 0.0001, ns: no significance.

Then, we explored the frequency and immunological features of TSPAN1^+^ B cells from PBMCs of AIH patients and HC by flow cytometry. The percentage of TSPAN1^+^ cells in B cells was significantly higher in AIH compared with HC. *t*-SNE analysis was performed to visually present the increment of TSPAN1 in CD19^+^ B cells (**Figures 4A, B**). We divided CD19^+^ B cells into two groups including TSPAN1^+^ cells and TSPAN1^-^ cells, to further explore the characteristics of TSPAN1^+^ B cells in AIH. Compared with TSPAN1^-^ cells, TSPAN1^+^ cells in AIH have higher expression of C-X-C chemokine receptor (CXCR) 3 (19.41% vs 5.73%, *P* < 0.001) and CXCR4 (66.56% vs 62.99%, *P* < 0.05), indicating enhanced chemotactic activity toward the liver. Antigen-presenting molecule CD86 (26.46% vs 13.57%, *P* < 0.001) was also highly expressed in TSPAN1^+^ B cells (**Figure 4C**). Furthermore, TSPAN1^+^CD19^+^ B cells could secrete higher levels of proinflammatory cytokines, including granzyme B (55.43% vs. 33.02%, *P* < 0.01), IFN-γ (52.82% vs. 27.68%, *P* < 0.01), and TNF-α compared to TSPAN1^-^ B cells (13.15% vs. 2.13%, *P* < 0.01). However, they also produced more TGF-β compared with TSPAN1^-^ B cells in AIH (49.36% vs. 30.12%, *P* < 0.01) (**Figure 4D**).

### 3.4 TSPAN1^+^ B cells presented a pro-inflammatory phenotype after *in vitro* stimulation

**Figure 5:**
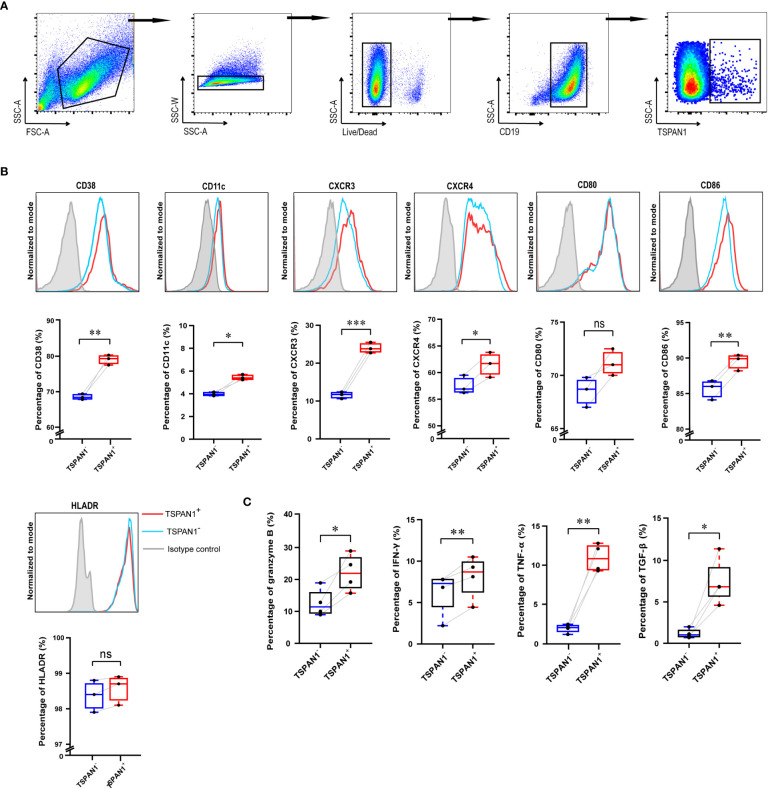
The immunological characteristics of TSPAN1^+^ cells after *in vitro* stimulation. **(A)** The gating strategy used for flow cytometry analysis of TSPAN1^+^ cells. **(B)** Histogram plot of surface markers of TSPAN1^+^ B cells and paired TSPAN1^-^ B cells (top row). Measurement of certain surface markers in paired TSPAN1^-^ cells and TSPAN1^+^ CD19^+^ cells were shown (bottom row). **(C)** The expression level of cytokines in paired TSPAN1^-^ cells and TSPAN1^+^ CD19^+^ cells. Paired Student *t*-test was used. *P < 0.05, **P < 0.01, ***P < 0.001, ns: no significance.

To explore whether TSPAN1^+^ B cells have an analogous immunological function after *in vitro* culture, CPG, CD40L, and IL-4 were utilized to stimulate CD19^+^ B cells isolated from PBMCs of healthy donors. Gating strategy was shown in **Figure 5A**. After stimulation, TSPAN1^+^ B cells expressed significantly increased CD11c (5.44% vs. 3.97%, *P* < 0.05), CD38 (79.03% vs. 68.53%, *P* < 0.01), CXCR3 (24.00% vs. 11.57%, *P* < 0.001), CXCR4 (61.53% vs. 57.53%, *P* < 0.05), and CD86 (89.50% vs. 85.63%, *P* < 0.01) compared with TSPAN1^-^ B cells (**Figure 5B**). We also observed that the expression of cytokines, including granzyme B (22.10% vs. 12.66%, *P* < 0.05), IFN-γ (8.08% vs. 6.15%, *P* < 0.01), TNF-α (10.93% vs. 1.89%, *P* < 0.01), and TGF-β (7.41% vs. 1.18%, *P* < 0.05) was higher in TSPAN1^+^ B cells (**Figure 5C**). These results were consistent with the immunological feature of TSPAN1^+^ B cells in peripheral blood from AIH.

### 3.5 TSPAN1^+^ cells infiltrated in the liver through CXCR3-CXCL10 interaction

**Figure 6:**
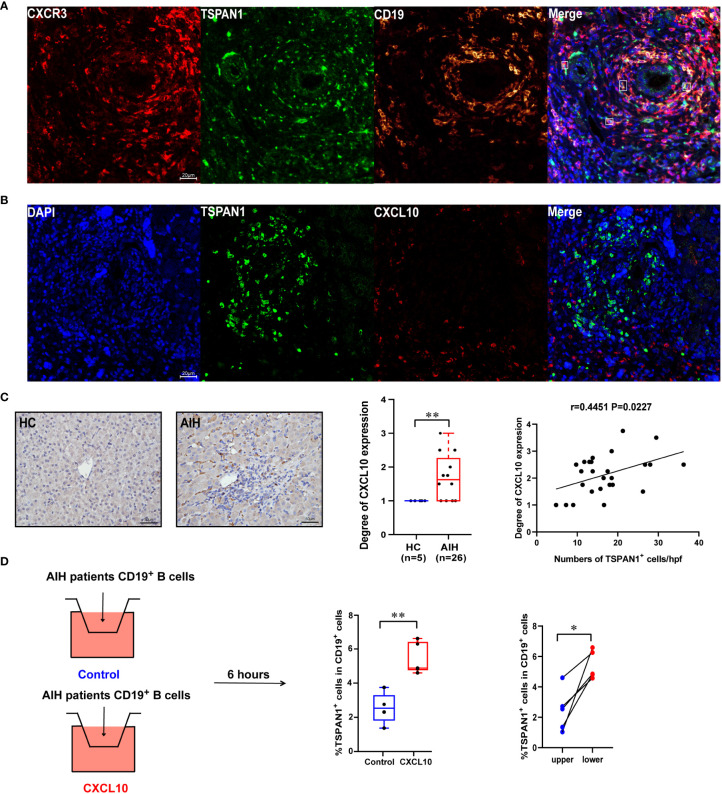
The expression of CXCR3 and CXCL10 in AIH. **(A)** Multiplex immunofluorescence staining of CXCR3 (red), CD19 (yellow), and TSPAN1 (green) in the liver of AIH patients. **(B)** Representative confocal staining of TSPAN1 and CXCL10 in the livers of AIH. **(C)** Immunohistochemical staining of CXCL10 in AIH and HC (×400). Correlation analysis of hepatic CXCL10 expression degree with the numbers of TSPAN1. **(D)** CD19^+^ B cells were placed in the upper chambers and complete medium was added in the lower chambers with rhCXCL10 (3 μg/mL) or not for 6 hours. The percentage of TSPAN1^+^ cells was measured by flow cytometry. Boxes represent the 25th–75th percentile of the distribution; the median is shown as a thick line in the middle of the box; whiskers extend to values with 1.5 times the difference between the 25th and 75th percentiles. *P < 0.05, **P < 0.01.

According to the results of flow-cytometric analysis, CXCR3 was highly expressed in TSPAN1^+^ B cells. As shown in **Figure 6A**, multiplex immunofluorescence staining was further confirmed the co-expression of CD19, TSPAN1, and CXCR3 in liver tissues from AIH. More interestingly, TSPAN1^+^ cells were closed to CXCL10 positive cells in the liver of AIH (**Figure 6B**). Since CXCL10 was the ligand for CXCR3, we detected the expression of CXCL10 in the liver tissues. The level of CXCL10 was significantly elevated in AIH compared with HC (*P* < 0.01). Besides, there is a correlation between the numbers of TSPAN1 and the expression of CXCL10 (r = 0.4451, *P* < 0.05) (**Figure 6C**). We next performed experiment to investigate the migration ability of TSPAN1^+^ B cells *in vitro*. CD19^+^ cells of AIH patients were isolated and stimulated for 24 hours and placed in the transwell chambers for 6 hours. As a result, a higher percentage of TSPAN1^+^ cells in CD19^+^ B cells were detected in lower chambers after adding rhCXCL10 compared with control group. Moreover, the percentage of TSPAN1^+^ cells in lower chambers was higher than that in paired upper chambers after adding rhCXCL10 (**Figure 6D**). These results suggested that the high expression of CXCL10 in the liver of AIH may contribute to the chemotaxis of TSPAN1-positive B cells.

### 3.6 Histological remission was accompanied with decreased TSPAN1 expression in AIH

**Figure 7:**
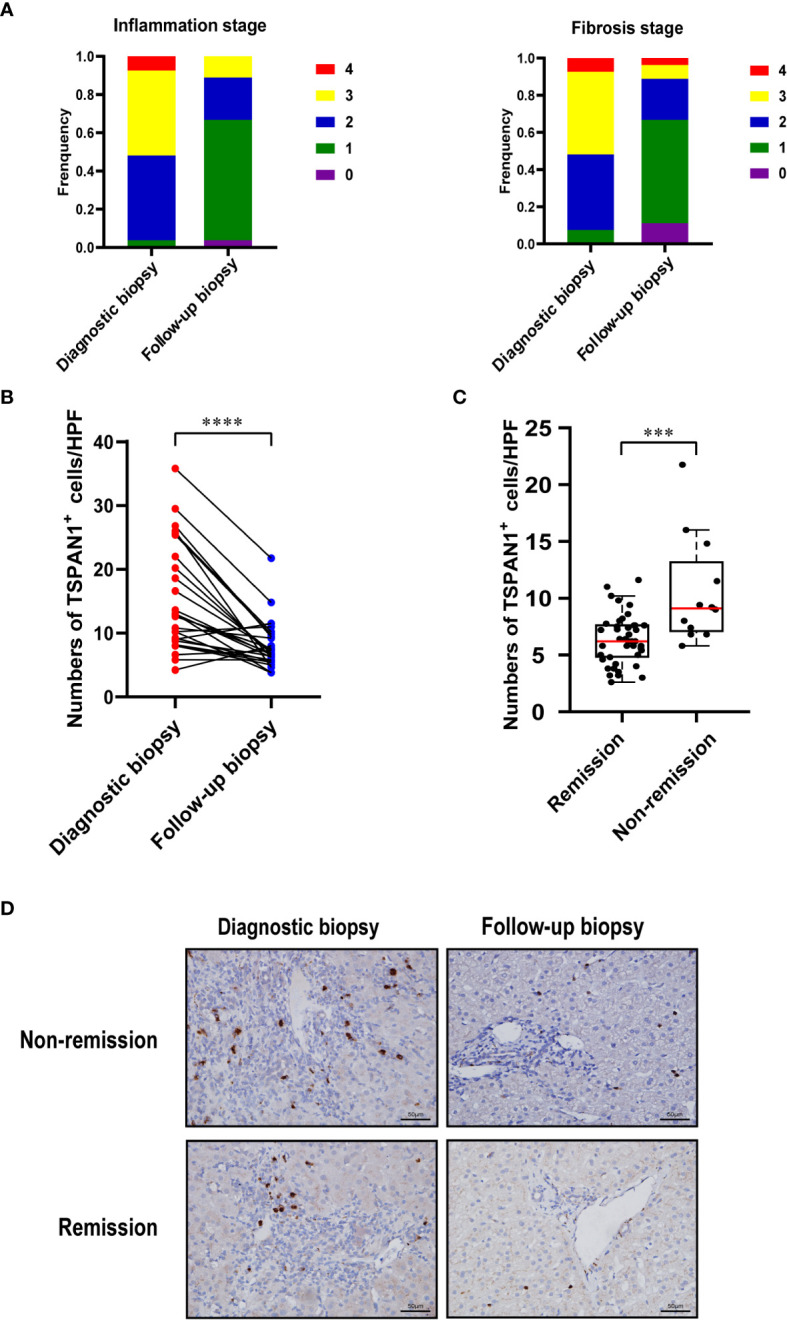
TSPAN1 expression was decreased after immunosuppressive treatment. **(A)** Hepatic inflammation and fibrosis stages were evaluated in diagnostic biopsies and follow-up biopsies in AIH patients. **(B)** Expression of TSPAN1 in paired diagnostic biopsies versus follow-up biopsies.**(C)** Expression of TSPAN1 in remission versus non-remission group. **(D)** Representative immunohistochemical staining of TSPAN1 in a diagnostic biopsy (left) and a paired follow-up biopsy (right) from non-remission (top) and remission group (bottom) (×400). ***P < 0.001, ****P < 0.0001.

Patients with AIH who had biopsies before and after treatments were enrolled (n =27). The paired biopsies of the liver showed that both hepatic inflammation and fibrosis were alleviated after treatments (**Figure 7A**). Immunohistochemical staining for TSPAN1 was performed. The results showed that the frequency of TSPAN1 was dramatically decreased in the liver tissues of AIH patients with treatment. To expand the follow-up biopsy cohort, we enrolled another 28 patients with a follow-up biopsy. 55 patients in total were divided into histological remission and non-remission. There were 43 patients in the remission (HAI < 4) group and 12 in the non-remission (HAI > 4) group. Interestingly, the frequency of TSPAN1 positive cells in the remission group was significantly decreased compared with that in the non-remission group (**Figures 7B–D**). These results suggested that TSPAN1^+^ cells may participate in the progression of AIH and were associated with histological remission.

## 4 Discussion

B cells have been demonstrated to be involved in the progression of AIH by producing autoantibodies, pro-inflammatory factors and functioning as APCs. In this study, we investigated the immunological feature of TSPAN1^+^ cells in AIH. Intriguingly, the numbers of TSPAN1^+^ cells were significantly increased in the livers of AIH and shared a close correlation with disease severity. According to confocal staining, TSPAN1^+^ cells were mainly CD19 positive B cells. However, these cells did not express CD27 or CD138, which may be naive B cells.

B cells can secrete antibodies, proinflammatory factors, and participate in antigen presentation, which are closely related to various diseases. Herein, compared with HCs, the proportion of TSPAN1^+^ cells in CD19^+^ B cells increased in the PBMC of AIH patients. It is also observed that TSPAN1^+^ cells secret more IFN-γ, TNF-α, and granzyme B, compared with TSPAN1^-^ cells. In addition, TSPAN1^+^ B cells expressed higher level of CD86, indicating their superior antigen-presentation ability.

Chemokine-receptor and ligand interactions have been identified as critical signals for immune cells recruitment. CXCR3 and CXCR4 were previously reported to be expressed by non-B lymphocyte immune cells and related to retention in the liver (26). Recent studies showed that CXCR3 and CXCR4 can also be expressed on B cells (27, 28). Our study demonstrated that CXCR3 was highly expressed on TSPAN1^+^ B cells. Interestingly, the chemokine CXCL10, a ligand of CXCR3, was detected and appear to contribute to the pathogenesis of various autoimmune disease. CXCL10 can bind to CXCR3 and regulate immune responses according to activation and recruitment of leukocytes. CXCL10 was also elevated in patients of AIH (9, 29) and observed highly expressed in liver tissues of patients by immunohistochemical staining. TSPAN1^+^ cells were adjacent to CXCL10 in the liver. Furthermore, the interaction between TSPAN1 and CXCL10 was further confirmed by transwell assay *in vitro*. CXCR3-CXCL10 interaction may contribute to the chemotaxis of TSPAN1^+^ B cells to the liver of AIH.

In this work, to better study TSPAN1^+^ cells *in vitro*, CPG, CD40L, and IL-4 were founded to enhance the expression of TSPAN1 in B cells. The liver is a unique immune regulatory organ characterized by complex immune activities triggered by a variety of immune cells, including B cells (30). CD40 signals together with other signals support the differentiation of B cells into plasma cells and further secrete various isotypes of antibodies. IL-4 is essential for B cell maturity and abundant in the liver (31). Combined with CD40 signaling, IL-4 promoted the proliferation of both circulating and tissue-resident B cells (32–34). Compared with peripheral blood, liver is enriched with various cytokines and lymphocytes, especially in autoimmune liver disease. Complex liver microenvironment provides a more proper condition for proliferation and aggregation of TSPAN1^+^ B cells, which partly explained why there were more TSPAN1^+^ B cells in the liver of AIH.

We found that the numbers of TSPAN1^+^CD19^+^ B cells were decreased in the liver of AIH patients after therapy. Furthermore, the frequency of TSPAN1^+^ cells was lower in the remission group compared with the no-remission group. These indicated that TSPAN1^+^ B cells may play an important role in the progression of AIH. Thus, our study provides a potential molecular target for the treatment of AIH.

In conclusion, this research found that TSPAN1^+^ B cells were elevated and may be involved in the pathogenesis of AIH. Overexpression of CXCL10 in liver may contribute to the chemotaxis of TSPAN1^+^ B cells. Besides, the frequency of TSPAN1^+^ cells was closely related to the remission of disease. TSPAN1 may be a potential target to alleviate AIH.

## Data availability statement

The original contributions presented in the study are included in the article/supplementary material. Further inquiries can be directed to the corresponding authors.

## Ethics statement

The studies involving human participants were reviewed and approved by the ethics committee of Renji Hospital, Shanghai Jiao Tong University. The patients/participants provided their written informed consent to participate in this study.

## Author contributions

QW, XM, and ZY designed and supervised the study. QW, RT, XM, and ZY acquired funding. YO and RC performed the experiments. YO, RC, QQ, and NC collected samples and clinical information. YO analyzed the data and drafted the manuscript. QW, XM, and ZY reviewed the manuscript. All authors contributed to the article and approved the submitted version.
